# COVID-19 and Cavitary Lesion in Lung

**DOI:** 10.7759/cureus.34098

**Published:** 2023-01-23

**Authors:** Avinash Vangara, Tara Hendrickson Rahmlow, Dedeepya Gullapalli, Sai Subramanyam Kommineni, Moeez Haroon, Subramanya Shyam Ganti

**Affiliations:** 1 Internal Medicine Residency Program, Appalachian Regional Healthcare, Harlan, USA; 2 Internal Medicine/Pulmonary Critical Care, Appalachian Regional Healthcare, Harlan, USA

**Keywords:** panton-valentine leukocidin (pvl), coronavirus disease 2019 (covid-19), mrsa (methicillin-resistant staphylococcus aureus), pulmonary embolism (pe), cavitary lung lesion

## Abstract

The clinical manifestations of coronavirus disease 2019 (COVID-19) caused by severe acute respiratory syndrome coronavirus-2 (SARS-CoV-2) are widespread, ranging from asymptomatic to critical illness with significant morbidity and mortality. It is widely known that individuals who have viral respiratory infections are more likely to develop bacterial infections. Throughout the pandemic, despite the fact that COVID-19 was thought to be the primary cause of millions of deaths, bacterial coinfections, superinfections, and other secondary complications played a significant role in the increased mortality rate. In our case, a 76-year-old male presented to the hospital complaining of shortness of air. Polymerase chain reaction (PCR) testing was positive for COVID-19 and cavitary lesions were discovered on imaging. Treatment was guided based on the results of bronchoscopy with bronchoalveolar lavage (BAL) cultures showing methicillin-resistant *Staphylococcus aureus *(MRSA) and *Mycobacterium gordonae*. However, the case was later complicated by the development of a pulmonary embolism after anticoagulants were held due to new onset hemoptysis. Our case highlights the importance of considering bacterial coinfection in cavitary lung lesions, appropriate antimicrobial stewardship, and close follow-up for full recovery in COVID-19 infections.

## Introduction

Coronavirus disease 2019 (COVID-19) caused by the severe acute respiratory syndrome coronavirus 2 (SARS-CoV-2) involves multiple organ systems, mainly lungs [1]. Secondary bacterial infections are very common in post-viral infections. Community-acquired staphylococcal pneumonia is usually seen in patients recovering from influenza with an increased risk seen in patients colonized with *Staphylococcus aureus* on their skin or the nares [1]. Active viral infections compromise local immunity within the respiratory tissues predisposing to the development of superinfection/coinfection causing necrotizing pneumonia leading to lung cavities [2]. However, it is uncommon to encounter a bacterial coinfection in patients with COVID-19 infection when compared to other viral pathogens like influenza [2]. Cavitary lesions are uncommon presentation and may indicate a superinfection/coinfection with bacterial/fungal pathogens, carry a poorer prognosis compared with other forms of pneumonia, and possibly indicate an increased risk for systemic complications including venous thromboembolism.

We present a unique case of a COVID-19 patient coinfected with methicillin-resistant *Staphylococcus aureus *(MRSA)/*Mycobacterium gordonae* that developed a cavitary lung lesion and eventually developed a pulmonary embolism.

## Case presentation

**Figure 1:**
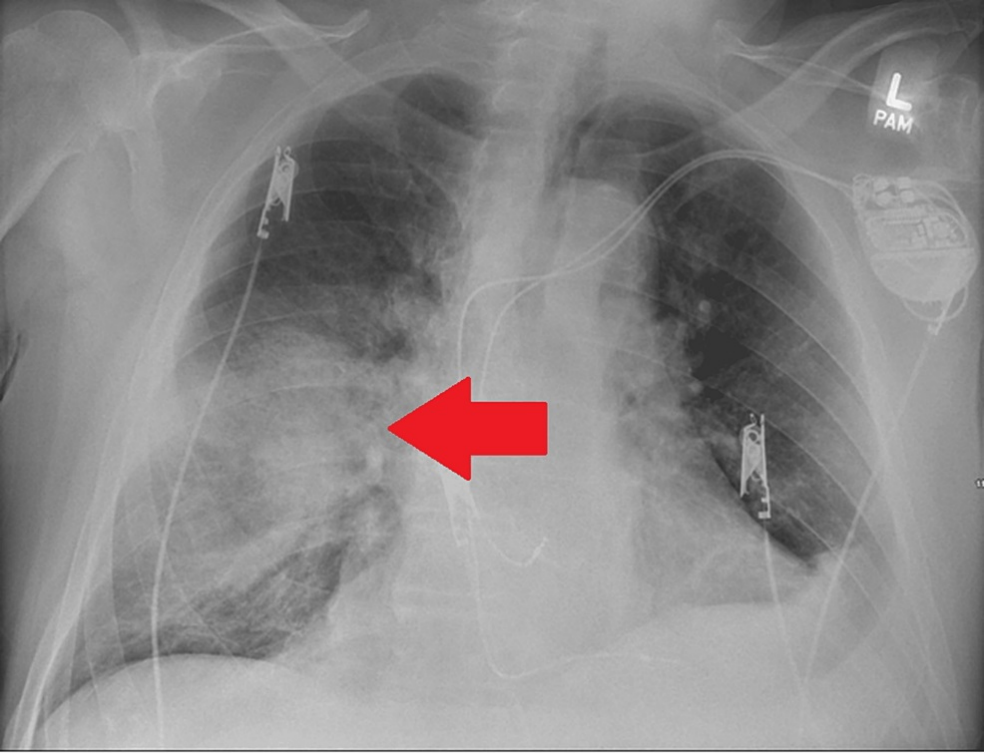
Posteroanterior chest radiograph showing perihilar consolidative opacity in the right lower lobe.

**Figure 2:**
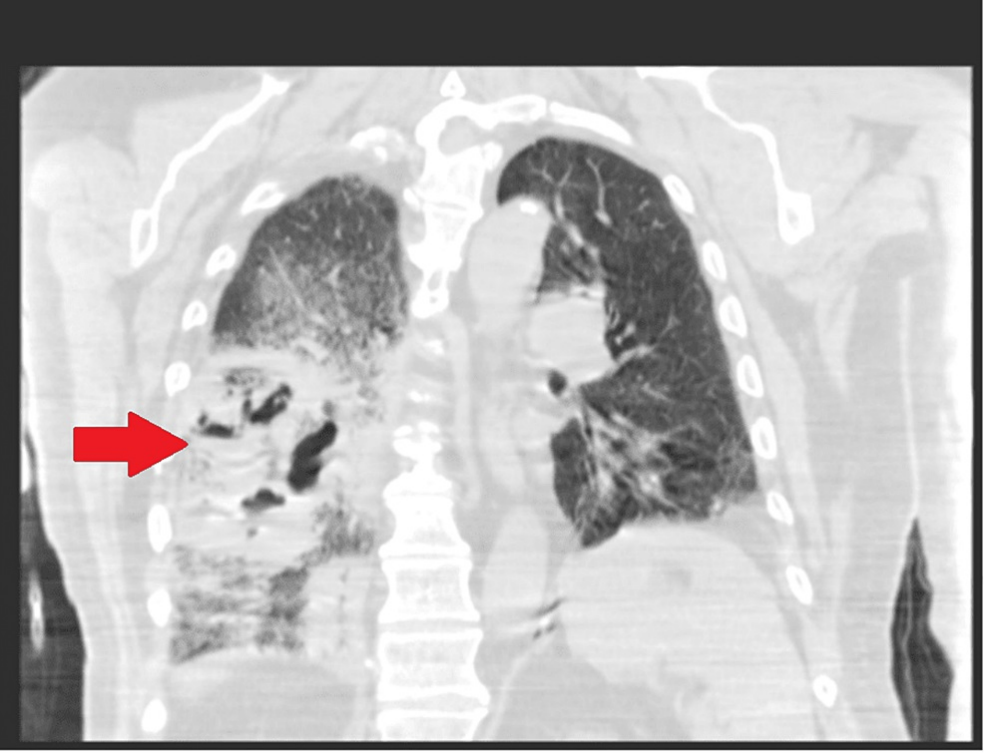
CT chest coronal view showing right lower lobe cavitary lesion.

A 76-year-old male presented to the emergency department (ED) with a three-day history of dyspnea, fever, and non-productive cough. His medical history was significant for chronic obstructive pulmonary disease (COPD) on home oxygen of 3 L/min, atrial fibrillation (anticoagulated with a novel oral anticoagulant {NOAC}, rivaroxaban), bilateral deep venous thromboses (DVT), and a 20-pack-year smoking history. The patient is on albuterol and Trelegy inhalers. He received COVID vaccine about a year ago. On presentation, the patient was found to be in acute respiratory failure with hypoxemia. On physical examination, the patient was noted to have diminished lung sounds, bilaterally, and diffuse expiratory wheezes, without crackles or rales. Initial work-up revealed an elevated white blood cell count and hyperbilirubinemia. Polymerase chain reaction (PCR) testing was positive for COVID-19. Subsequent laboratory tests were significant for an elevation in the COVID-19-associated inflammatory markers like D-dimer, ferritin, lactate dehydrogenase, and C-reactive protein. Telemetry showed an irregular rhythm and a subsequent EKG confirmed atrial fibrillation with rapid ventricular response (RVR). The patient was initially treated with a diltiazem drip but as the heart rate was not controlled, later switched to an amiodarone drip. The patient’s home dose of rivaroxaban was continued for the management of atrial fibrillation and as a means for venous thromboembolism (VTE) prophylaxis. Chest x-ray on admission revealed a perihilar consolidative opacity in the right lower lobe (Figure 1). Follow-up non-contrast computed tomography (CT) chest confirmed a right lower lobe cavitary lesion (Figure 2).

The patient was started on empiric antibiotic coverage with piperacillin-tazobactam and linezolid. The patient was requiring high oxygen flow rates to maintain his saturation above 88%. He was requiring a Vapotherm of 30 L/min at one point. Remdesivir and dexamethasone were started for COVID-19-related respiratory support. On hospital day two, the patient developed multiple episodes of hemoptysis with an associated drop in the hemoglobin from 16.8 g/dL on admission to 14.4 g/dL, with a normal platelet count of 196x10^3^/μL, and anticoagulation was withheld. A bronchoscopy with bronchoalveolar lavage (BAL) showed blood-tinged mucus plugs in the right mainstem bronchus as well as the right upper and lower lobes. The bronchoscopy also found a collapse of the superior and posterior segments of the right lower lobe, seemingly due to extrinsic compression. Low-molecular-weight heparin was started after BAL for VTE prophylaxis. Sputum culture and BAL from the right lower lobe grew methicillin-resistant *Staphylococcus aureus *(MRSA) and the patient’s antibiotic regimen was narrowed to monotherapy with linezolid. Sensitivity testing showed a minimum inhibition concentration (MIC) of 1 μg/mL for vancomycin. As the patient was already on linezolid and also as the study by Tong et al. has shown linezolid has better cure rates, the antibiotic selection was narrowed down to linezolid. Sputum culture and BAL results did not show any malignant cells. Blood cultures were negative. The patient improved clinically, with no additional episodes of hemoptysis, and was discharged after staying in the hospital for nine days. He was weaned to his baseline oxygen requirement before discharge. He was instructed to resume his home dose of rivaroxaban, barring any additional hemoptysis.

Two days later, the patient returned to the ED with complaints of an acute onset of worsening dyspnea and nausea. Laboratory studies revealed a significant elevation in the D-dimer (7.84 mg/L FEU), alongside persistent leukocytosis. Venous duplex scan of lower extremities showed bilateral DVTs. Contrast-enhanced CT chest confirmed a pulmonary embolus in the distal right main pulmonary artery extending into the right upper lobe branches. Further investigation revealed that the patient had failed to resume his home dose of rivaroxaban, in fear of inciting further episodes of hemoptysis. The patient was educated on the importance of continuing anticoagulation therapy, along with completing the antibiotic course with linezolid, and was subsequently discharged home the following day. A follow-up appointment was scheduled with pulmonology for the continued management of his right lower lobe cavitary lesion.

The acid-fast bacterial culture of the specimen retrieved from the BAL result was positive for *Mycobacterium gordonae* (*M. gordonae*), however, this result wasn't finalized until four weeks post-discharge. The patient had significant clinical improvement with linezolid. Treatment for *M. gordonae *was not started based on infectious disease recommendations as *M. gordonae *is known to be the least harmful species of mycobacteria, and its isolation is frequently viewed as a contaminant.

## Discussion

Although significant differences may be seen on CT scans from COVID-19 patients, typical characteristic radiographic features include multifocal and bilateral ground-glass opacities in a predominantly peripheral distribution [1]. CT findings of pleural effusion, pericardial effusion, and pulmonary cavitation are uncommon and should prompt further investigation in these patients given the increased rates of morbidity and mortality seen with viral-bacterial coinfections.

Cavitation is a complication that may occur in necrotizing pneumonia and consists of an air-filled space developing within areas of established pulmonary consolidations. Usually seen in fungal, parasitic, bacterial, or neoplastic pathogenesis, pulmonary cavitating lesions develop as a direct result of liquefactive necrosis within these consolidations [1]. Viral pneumonia-causing cavitation is relatively uncommon [2]. *Staphylococcus aureus*, a known cause of community and hospital-associated infections was a major cause of mortality during the 1918 and 2009 influenza pandemics and is often seen as a secondary infection or superinfection but rarely seen as a coinfection in immunocompetent patients such as in this case [3]. Researchers have discovered that some MRSA strains contain genes for the Panton-Valentine leucocidin (PVL) toxin which has been shown to be responsible for many of the severe clinical symptoms of infection including severe necrotizing pneumonia and necrotic lesions of the skin and soft tissue [4]. According to Langford et al. study, bacterial coinfection was only reported in 3.5% (95% CI: 0.4-6.7%) of patients, and secondary infection in 14.3% (95% CI: 9.6-18.9%) of patients with COVID-19 [5]. Although *M. gordonae* is typically regarded as a contaminant, there have been reports of clinically significant disease [6]. The presence of clinical symptoms and radiographic abnormalities are required for the diagnosis [7] with the most common symptomatic manifestations being cough, weight loss, dyspnea, hemoptysis, and fever [8] while radiographic findings may include pulmonary nodules, cavities, infiltrates, and consolidation [6].

As described in the study by Bhopalwala et al., a case of invasive pulmonary aspergillosis (IPA) that occurred after treatment of COVID-19 infection shows an increase in the probability of complications like IPA and ARDS with immunosuppression drug use [9]. Our patient developed hemoptysis one day after admission which is commonly seen in patients with necrotizing pneumonia. Based on autopsy reports, the Selvaraj and Dapaah-Afriyie study concluded that hemoptysis may be secondary to diffuse alveolar damage, intraalveolar hemorrhage, or necrosis of parenchyma [10]. In one systematic review, 22% of ante- or postmortem dissected lungs displayed macroscopic hemorrhagic changes. Histopathologically, alveolar hemorrhage was seen in 33% and partial hemorrhagic necrosis in 0.3% of cases [11]. COVID-19 patients requiring high oxygen concentrations for a prolonged time have an increased risk for pneumothorax/pneumomediastinum as barotrauma risk is increased with diffuse alveolar damage and poor lung compliance [12].

Empiric broad-spectrum antibiotic therapy is not recommended in COVID-19-positive patients unless a bacterial coinfection is highly suspected or proven with positive cultures. A minimally invasive technique, bronchoalveolar lavage (BAL) is commonly used to obtain a specimen of the lower respiratory tract. In the diagnosis of infectious diseases, BAL has advanced significantly with the development of new assays for microbial products (such as galactomannan for invasive fungal disease), immunostaining for organisms (such as direct fluorescent antibody for pneumocystis), and nucleic acid amplification tests for particular microbes. While differential cell count profiles and ancillary testing on BAL fluid are not diagnostic or specific in and of themselves, in the appropriate clinical context, these findings may be the deciding factor in reaching a specific diagnosis and subsequently guiding treatment [13].

According to the most recent Infectious Diseases Society of America (IDSA) MRSA infection clinical practice guidelines, intravenous vancomycin or oral/intravenous linezolid is recommended for the treatment of MRSA pneumonia [14]. Studies have shown that when assessing the clinical efficacy of vancomycin and linezolid, patients treated with linezolid had a higher rate of microbiologic success (81.9% vs 60.6%) and clinical cure (57.6% vs 46.6%), as well as lower incidence of nephrotoxicity (8.4% vs 18.2%) [15]. Linezolid is also known to have high penetration into the lung tissue and causes inhibition of PVL toxin production thus preventing additional cavitation [15].

*M. gordonae *is generally considered a containment that is usually found in soil and water. The significance of *M. gordonae *isolation should be determined in conjunction with the clinical presentation. Susceptibility testing is not routinely recommended [16], however, when pathogenicity is determined, antimicrobial agents most consistently active in vitro include ethambutol, rifabutin, clarithromycin, linezolid, and fluoroquinolones [7]. BAL culture report resulted after four weeks of discharge of the patient from the hospital. As the patient had significant clinical improvement by this time, it was determined to be as containment and was decided not to treat after discussing it with an infectious diseases specialist.

For non-critically ill patients hospitalized with COVID-19, a high index of suspicion is necessary for detecting thromboembolism, especially when the patient presents with acute abdominal pain, chest pain, or lower extremity pain [17,18]. Therapeutic-dose heparin appears beneficial, with a high probability of reducing the need for organ support and the progression to intubation and death, regardless of D-dimer results [19]. Our patient was started on low-molecular-weight heparin. Along with anticoagulation, heparin has a role in the disruption of clot production as well as ameliorating the inflammatory effects of thrombin. It has antiinflammatory properties including antagonizing cytokines [20]. Up to one-third of COVID-19 patients experience thrombotic episodes, which are linked to more severe illness and higher mortality [21]. Thrombotic signs impact both the venous and arterial sides. COVID-19 is associated with an increased risk of thrombosis and microangiopathic problems [22]. Two days post-discharge, our patient presented with worsening dyspnea, and labs revealed an elevated D-dimer level of 7.84 mg/L FEU. According to the meta-analysis, a systematic review of PE, and DVT in COVID-19, D-dimer levels greater than 500 µg/L and greater than 1000 µg/L showed high sensitivity (96% and 91%, respectively) but low specificity (10% and 24%, respectively) in the diagnosis of PE in patients with COVID-19 [23]. Higher cutoff values than those showed reduced sensitivity, limiting the usage of D-dimer as a screening tool. Compared with a fixed D-dimer cutoff of 500 µg/L, the combination of pretest clinical probability assessment with age-adjusted D-dimer cutoff was associated with a larger number of patients who could be considered ruled out with a low likelihood of subsequent clinical venous thromboembolism [24]. Given our patient’s elevated D-dimer and high clinical probability of a pulmonary embolism, a chest CT angiogram was performed which showed a distal right main pulmonary artery thrombosis as well as a cavity in the right lower lobe.

The retrieved literature on the epidemiology of MRSA lung infection in COVID-19 patients revealed significant variability, with relative prevalence ranging from 11% to 65% when *S. aureus *was taken into account and from 2% to 29% when all other bacteria were taken into account [25]. It should be noted that although MRSA was frequently the etiology of community-acquired bacterial pneumonia in COVID-19 patients, all of this also likely reflects the local microbiological epidemiology [25]. Increasing our capacity to quickly identify the pneumonia-causing agents and their critical resistance in COVID-19 patients, it is essential to determine appropriate empirical therapy while also avoiding antibiotic overuse in accordance with antimicrobial stewardship principles.

## Conclusions

This case highlights the occurrence of pulmonary cavitation seen in COVID-19-infected patients. Although there is a wide range of pulmonary disease conditions that frequently appear as cavities, cavitation in viral pneumonia is rare unless there is a coexisting infection. Given the documented high rate of morbidity and mortality seen with bacterial-viral coinfections in previous pandemics, it is essential to have a high clinical suspicion when suspicious pathology is seen on imaging. Chest CT is the mainstay of imaging and early utilization should be done in patients with suspected cavitary lesions. When treating individuals with COVID-19 and necrotizing pneumonia, it is important to take into account adding antistaphylococcal antimicrobial drugs with good lung penetration and/or antitoxin production, such as linezolid. Secondary complications like pulmonary embolism or thrombus should be considered and prompt evaluation with contrast-enhanced CT should be utilized when evaluating COVID-19-positive patients, especially those with markedly elevated D-dimer levels and high clinical suspicion.
